# Supplementary figures and images for: The impact of the SARS COV-2 pandemic on pediatric accesses in ED: A Healthcare Emergency Information System analysis
Source: PLoS One. 2022 Aug 5;17(8):e0272569. doi: 10.1371/journal.pone.0272569 (PMC9355200; doi:10.1371/journal.pone.0272569)

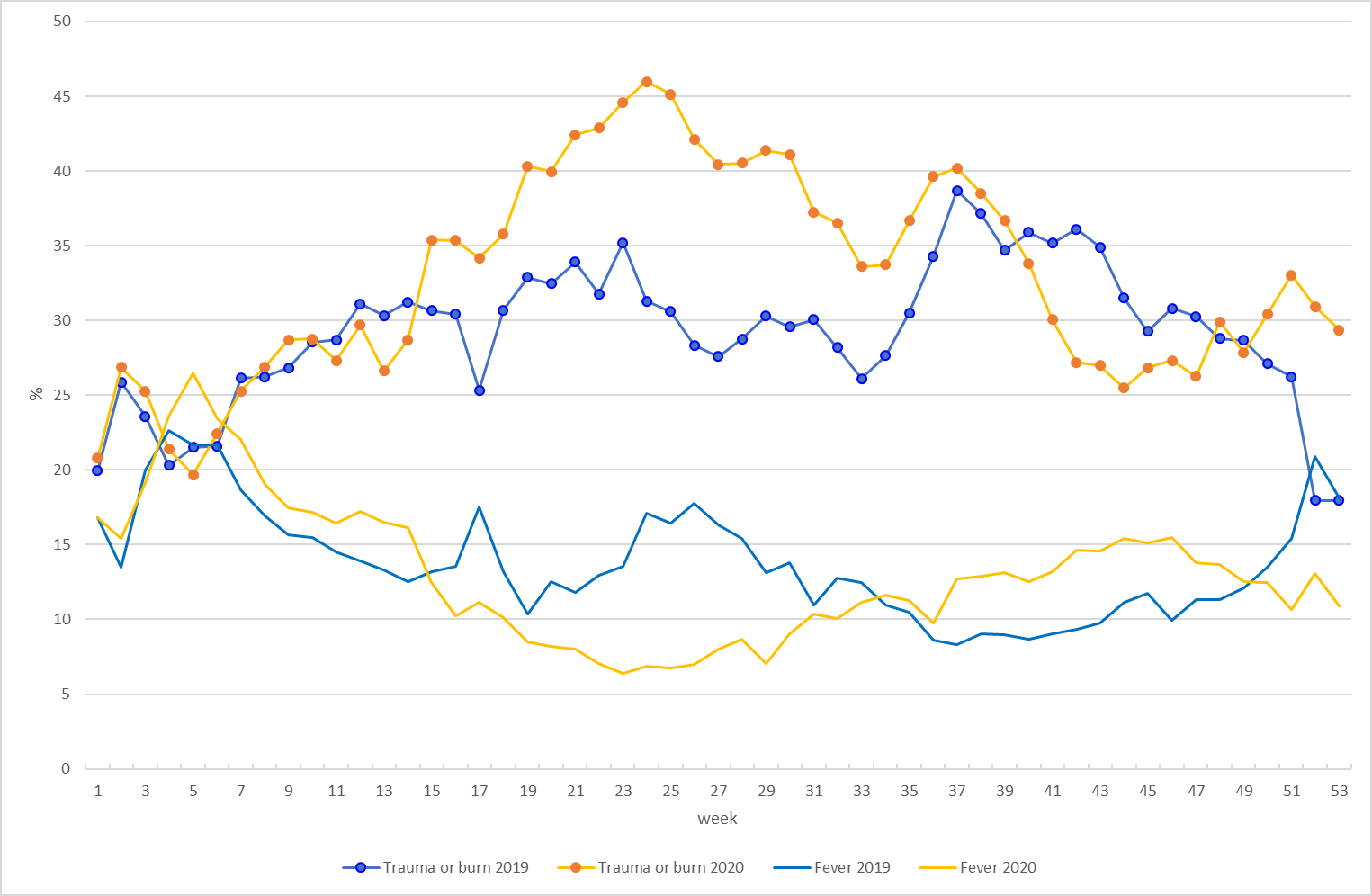

Supplement: S1 Fig — Yellow and blue lines with dots indicate the percentage of pediatric ED accesses for trauma and burn in 2020 and 2019 respectively; yellow and blue lines without dots indicate the percentage of pediatric ED accesses for fever in 2020 and 2019 respectively. (TIF) [file pone.0272569.s002.tif]
