# Supplementary material for: The impact of the SARS COV-2 pandemic on pediatric accesses in ED: A Healthcare Emergency Information System analysis
Source: PLoS One. 2022 Aug 5;17(8):e0272569. doi: 10.1371/journal.pone.0272569 (PMC9355200; doi:10.1371/journal.pone.0272569)
Supplement: Attachment — Submitted filename: Response to Reviewers.docx [file pone.0272569.s004.docx]

Dear Editor,

We would like to thank the reviewers for the appreciation of our work and for the interesting comments to our manuscript. The comments were useful to improve the clarity of the paper.

Listed below our answers to the comments of the referees.

We really hope that our changes will satisfy the editorial needs.

**POINT BY POINT RESPONSE TO THE COMMENTS**

**Comments to the Author**

**Reviewer #1: All comments and feedback have been rightly addressed. I thank the authors for taking these suggestions to heart and assimilating them to ensure that sound data is out out there for the scientific community and beyond.**

**Reviewer #3: This is a limited descriptive study which clearly shows that there was an impact on ED attendances in children in the Lazio region of Italy during 2020. This is shown as a comparison to 2019, and does not take into account any possible trends.
Numerous studies have now shown that the pandemic affected ED attendances. This is another study which adds to this evidence, and has the advantage of showing that the impact continued into 2020 beyond the first lockdown.

I do not feel able to comment on the extensive comments by Reviewer 2 on data access. I am surprised that ethical approval is not required for use of deidentified data – I have to obtain approval in the UK, although the process is relatively quick.**

***Answer:*** *In Italy is not necessary the approval by an ethical committee when the analysis are based on anonymized data from Health Information Systems.*

**I do think that there needs to be tightening up of language the paper, which is clearly comprehensible, but some of the tenses and word choice is surprising.**

***Answer****: the language has been revised.*

**I would like to see more information in the legend/title of the figures and less in the text.
For example, the different colours over time in Figures 1,2 and 4 should be explained in the Figure legend. I am not sure what they correspond to.**

***Answer****: The different colours of the time represent the four phases of the pandemic in 2020: grey= pre-lockdown, orange=lockdown, light blue=post lockdown and yellow= the second wave. Thanks for your suggestion we have integrated this information in the figure legends.*

**Figure 3 is an unusual presentation of data and I do not think it adds.**

***Answer:*** *Figure 3 is a modified age pyramid that compared ED accesses in 2019 and 2020 by age classes, instead of male and female population. In our opinion is useful to understand the different impact of the pandemic on ED accesses by age classes.*

**The methods should emphasise that this is a descriptive paper.**

***Answer:*** *We modified the text reporting that this is a descriptive study both in the “Material and Methods” paragraph and in the “Discussion” one.*

**I would like to see a justification of the age groups which I am sure are reflective of Italian systems, but seem irregular to a non-Italian reader.**

***Answer:*** *Age groups reflect the Italian education from the nursery school to the high school. Thanks to your suggestion, we added this information in the methods paragraph.*

**Results in Table 1 include a p-value but it is not clear in the methods which statistical test was used to assess this change.
Related to this on line 168 you say: we noticed that there are none important differences between 2019 166 and 2020 during the “pre-lockdown” period (-0.4% for all ages)’ despite a p-value of <0.001. I am not surprised by the significant p-value, but it is odd to class something as unimportant when you have identified it as significant statistically.**

***Answer:*** *Thanks for your note, actually the difference between ED accesses of 2019 and 2020 for the “pre-lockdown” period is not significant, it was an oversight. We have modified the table reporting the exact p-value.*

**Line 196 – increament – this is not a word – increment does not really work in this sentence – increase would work well here.**

***Answer:*** *The sentence has been revised.*

**The Figure legends should include %Var children if this is what the grey bars are.**

***Answer:*** *The figure legends have been modified as requested.*

**The discussion is fine, but does not add very much to add to our understanding of the challenges and impact on children’s health during lockdown.**
 **I enjoyed the final paragraph of the conclusion.**
